# The impact of randomized controlled trials on the management of anterior shoulder instability: A bibliometric and altmetrics analysis

**DOI:** 10.1002/ksa.70333

**Published:** 2026-02-16

**Authors:** Amr AlMasri, Rohaan Syan, Danielle Dagher, Ashraf Hantouly, Khalid Alkhelaifi, Moin Khan

**Affiliations:** ^1^ Department of Medicine McMaster University Ontario Hamilton Canada; ^2^ Department of Medicine University of Ottawa Ontario Ottawa Canada; ^3^ Division of Orthopaedics, Department of Surgery McMaster University Ontario Hamilton Canada; ^4^ Aspetar Orthopaedics and Sports Medicine Hospital Doha Qatar

**Keywords:** altmetric analysis, anterior shoulder, bibliometric analysis, randomized controlled trials, shoulder instability

## Abstract

**Purpose:**

To perform a bibliometric and altmetric analysis of randomized controlled trials (RCTs) in anterior shoulder instability (ASI) management through a citation and altmetrics analysis.

**Methods:**

A complete search of MEDLINE, EMBASE and CENTRAL databases was conducted from inception to 17 July 2025 for RCTs assessing surgical management of ASI. Citation metrics were obtained from Clarivate Web of Science Database on 16 August 2025, and Altmetrics data were extracted from Altmetrics.com on 5 September 2025. Univariate regression models and a one‐way analysis of variance were used to explore correlations between scholarly impact and various study characteristics.

**Results:**

A total of 24 studies consisting of 1559 participants were included. Studies were published between 1999 and 2024. Eleven countries were represented, with most studies originating from Canada (*n* = 5). The average impact factor was 4.3 (range 0.3–5.4), and the median citation density was 4.90 citations per year (range 1.0–19.3). The average altmetrics attention score was 10.8 (range 1–25). The median scholarly composite score density (SCSD) was 6.75 mentions per year (range 0.05–24). Regression analysis demonstrated fair and significant correlation to the first author's *H*‐index for both citation density (*r* = 0.44, *p* = 0.030) and SCSD (*r* = 0.41, *p* = 0.047). All other variables were non‐significant but had high effect sizes. Risk of bias (RoB) scores were found to be fairly correlated with citation density (r = 0.43, p = 0.036).

**Conclusions:**

The bibliometric and altmetric impact of RCTs assessing surgical management of ASI is wide and varied across many journals of variable impact factors. Despite high effect size, only the first author's *H*‐index was correlated with higher citation and SCSD, while RoB score was fairly correlated with a higher citation density, posing the question of whether an author's preestablished reputation or the robustness of an RCTs' design to control for bias can have an impact on the dissemination of academic information.

**Level of Evidence:**

Level V, expert opinion.

**Abbreviations**
ASI: anterior shoulder instability
RCTs: randomized controlled trials
RoB: risk of bias
SCSD: scholarly composite score density
SqrtIF: square root impact factor

## INTRODUCTION

Anterior shoulder instability (ASI) is one of the most common shoulder pathologies encountered in young and active populations, particularly athletes involved in contact or overhead sports, and is typically the result of a trauma‐induced glenohumeral dislocation [6, 11, 63]. Management strategies for ASI have evolved to address both soft tissue and bony pathology, with surgical intervention increasingly favoured for young, high‐risk patients due to lesser risk of recurrence [34, 59].

As it stands, treatment options range from arthroscopic Bankart repair to bone augmentation procedures such as the Latarjet, with ongoing debate regarding optimal surgical approach [16]. While numerous cohort and case series studies have contributed to the evidence base, randomized controlled trials (RCTs) remain more difficult to perform in orthopaedic surgery, including in the context of ASI due to conflicts with blinding and potential expertise bias [42]. Given their ability to minimize bias and provide high‐level evidence, RCTs are considered the gold standard for guiding clinical practice, yet logistical and methodological challenges often limit their conduct in surgical fields [43].

Bibliometric indicators have long served as a proxy for the academic impact of published studies and have helped identify which trials most strongly influence practice and guideline development. Some of the widely used metrics include a study's cumulative citation rate, the publishing journal's impact factor or the first author's H‐index. Bibliometric analyses have been employed across multiple orthopaedic subspecialties to evaluate research trends, highlight influential publications and identify gaps in the literature [17, 26]. These metrics help inform users about the academic influence a particular study has had within the area being studied but are limited by their limited academic use and by the fact that they take years to accumulate and can even be influenced or manipulated [25, 35]. Other forms of information dissemination have recently emerged as well, particularly with the ever‐increasing popularity of social media platforms such as X (formerly Twitter) and Facebook. The ease of access to those platforms has opened the gate to the creation of new alternative metrics that have since become easily available and can showcase an article's reach and influence over a bigger, more public social circles [1].

As such, understanding which trials shape the field, and whether citation or altmetric activity reflects study quality or clinical relevance, may provide insights into how evidence disseminates and guide future research priorities. Therefore, the purpose of this study was to perform a bibliometric and altmetrics analysis of RCTs investigating the management of ASI. By characterizing the most highly cited trials, analyzing patterns of citation and altmetric activity, and evaluating potential associations with study characteristics, this work aims to identify the current drivers of evidence in ASI and areas where further high‐quality research is needed.

## METHODS

### Search strategy

This study conducted a systematic review based on the guidelines set out by the Cochrane Handbook and PRISMA 2020 Guidelines for systematic reviews [28, 51]. A preliminary search of the literature was conducted, followed by the development of a comprehensive search strategy based on two main concepts: Randomized control trials (RCTs) and the management of ASI (Table 1). This study was not registered in a systematic review database.

**Table 1:** Search strategy employed with relevant keywords used.

OVID search terms for MEDLINE, EMBASE and CENTRAL
1. anterior shoulder dislocation.ti,ab,kf. 2. anterior shoulder instability.ti,ab,kf. 3. glenohumeral instability.ti,ab,kf. 4. exp shoulder dislocation/ 5. shoulder subluxation.ti,ab,kf. 6. exp Joint Instability/ 7. (shoulder adj3 (instabil$ or dislocat$ or subluxat$)).ti,ab,kf. 8. (glenohumeral adj3 instabil$).ti,ab,kf. 9. randomized controlled trial.pt. 10. controlled clinical trial.pt. 11. randomized.ti,ab,kf. 12. randomly.ti,ab,kf. 13. 1 or 2 or 3 or 4 or 5 or 6 or 7 or 8 14. 9 or 10 or 11 or 12 15. 13 and 14

### Eligibility criteria

Inclusion criteria were: (1) RCTs, (2) evaluating ASI, subluxation, or dislocation, (3) containing at least one surgical intervention arm or examining at least one surgical technique and (4) available in English. Exclusion criteria were: (1) incomplete/ongoing RCTs, (2) pilot/feasibility RCTs, (3) did not have a surgical management protocol, or a protocol that only involved perioperative analgesics, or perioperative adjuncts (such as platelet‐rich plasma, splinting or physiotherapy). After developing the search strategy, MEDLINE, EMBASE and CENTRAL databases were electronically searched from database inception to 17 July 2025.

### Study screening

Two independent authors (A.A. and R.S.) conducted the title/abstract and full‐text screening of all the available articles. Conflicts were resolved by discussion and consensus among the two authors. The inter‐rater reliability was measured at both the title and abstract as well as full‐text screening stages using Cohen's kappa (*κ*) coefficients. The agreement was categorized a priori, as per Landis and Koch, with a value of 0.81–1.0 representing near‐perfect agreement, 0.61–0.80 representing substantial agreement, 0.41–0.60 representing moderate agreement, 0.21–0.40 representing fair agreement and 0.00–0.20 representing slight agreement [36].

### Data extraction

The two authors (A.A. and R.S.) independently extracted relevant study characteristics into a data extraction sheet designed a priori in Google Sheets (Google Sheets, Google LLC). Discrepancies were resolved through discussion among the authors. The extracted data included study characteristics (i.e., author, journal, year of publication, country of correspondence, corresponding author and PMID), trial characteristics (i.e., number of participating sites and countries, recruitment period, number and characteristics of the intervention/control group, power analysis, patient‐reported outcome measures [PROMs], radiographic outcomes and other reported outcome measures such as complications, recurrence rate, range of motion, strength testing measures or study specific functional outcome measure assessments/scores), participant characteristics (i.e., study population, inclusion and exclusion criteria, % female, mean age and mean follow‐up period), and study outcomes. Bibliometric data (i.e., journal impact factor, number of citations and first and last author *H*‐index) were obtained from Clarivate Web of Science Database on 16 August 2025 as per previous studies [1, 7, 18, 24]. Altmetrics data (i.e., Altmetrics attention score, number of mentions on X, Facebook, news, blogs, policy and Wikipedia, number of Mendeley readers and number of X users reached) were collected from Altmetrics.com on 5 September 2025 as per previous studies [3, 5, 22, 38]. In addition, all studies were categorized into one of four categories that were used for analysis of variance (ANOVA) analysis: (1) assessing outcomes following minor changes to surgical technique; (2) assessing outcomes following major changes to surgical technique; (3) assessing surgical versus non‐surgical management and (4) comparing two surgical procedures. The categorizations were used for ANOVA analysis.

### Risk of bias (RoB) assessment

The two independent authors (A.A. and R.S.) assessed the RoB of included studies using the revised Cochrane risk‐of‐bias tool for RCTs (RoB‐2) [29, 44, 60]. The following methodological domains were assessed: randomization process, deviations from the intended interventions (effect of assignment to intervention), missing outcome data, measurement of the outcome, and selection of the reported result. Each domain was rated as ‘Low risk’, ‘High risk’ or ‘Some concerns’ with a study‐level RoB judgement being given according to the criteria provided by the RoB‐2 tool. Disagreements were resolved by consensus between the two authors. Scores were then converted to a score out of 5 (0–5), with a study scoring a point for every ‘Low Concern’ domain it achieved during its assessment, meaning that a higher RoB score euqalled less bias concern a study had.

### Statistical analysis

Before the analysis was conducted, a number of other metrics were calculated based on extracted data. Citations per year (also referred to as citation density) was defined as the number of citations a study had divided by the number of years since publication. Scholarly composite Score was calculated as the total number of citations plus the total number of online mentions (The sum of the number of mentions on X, Facebook, news, blogs, policy and Wikipedia). Finally, the Scholarly composite score per year (also known as Scholarly composite score density) was calculated as the Scholarship Composite Score divided by the number of years since publication.

Statistical analyses were performed using IBM SPSS Statistics for Windows (Version 27). Variables included were journal impact factor, years since publication, category assignment, number of PROMs, total number of outcome measures, number of trial sites, mean follow‐up time, total sample size, first and last author *H*‐index and RoB score.

The Shapiro–Wilk test was used to assess the normality of the variable distribution. Variables that violated the test were transformed via the root mean square [1, 52, 53]. Out of the listed variables, only the journal impact factor violated the normality test and as such was transformed to the square root of impact factor (SqrtIF). Correlations between citation/Scholarly composite score density and the other aforementioned variables were explored using univariate linear regression models that utilized the Pearson coefficient to test direct correlations. This analysis was modelled after methodologies used in other bibliometric analyses [1, 17, 33]. In addition, a one‐way ANOVA comparing study categories was performed on citations per year, sample size, SqrtIF, number of PROMs, total number of outcome measures, number of trial sites, follow‐up means, total sample size, RoB scores, First and Last author *H*‐Indexes, and Scholarly Score per year by study category. A *p* value of ≤0.05 was considered statistically significant. Correlation coefficient (*r*) strength was defined as Very Strong if *r* greater than or equal to 0.8, Moderately strong if between 0.6 and 0.8, Fair if between 0.3 and 0.5 and Poor if less than 0.3 [14]. Effect size was calculated in the ANOVA and represented by *η* ^2^, where *η* ^2^ = 0.01 indicates a small effect, *η* ^2^ = 0.06 indicates a medium effect and *η* ^2^ = 0.14 indicates a large effect [55].

## RESULTS

### Study characteristics and RoB assessment

**Figure 1:**
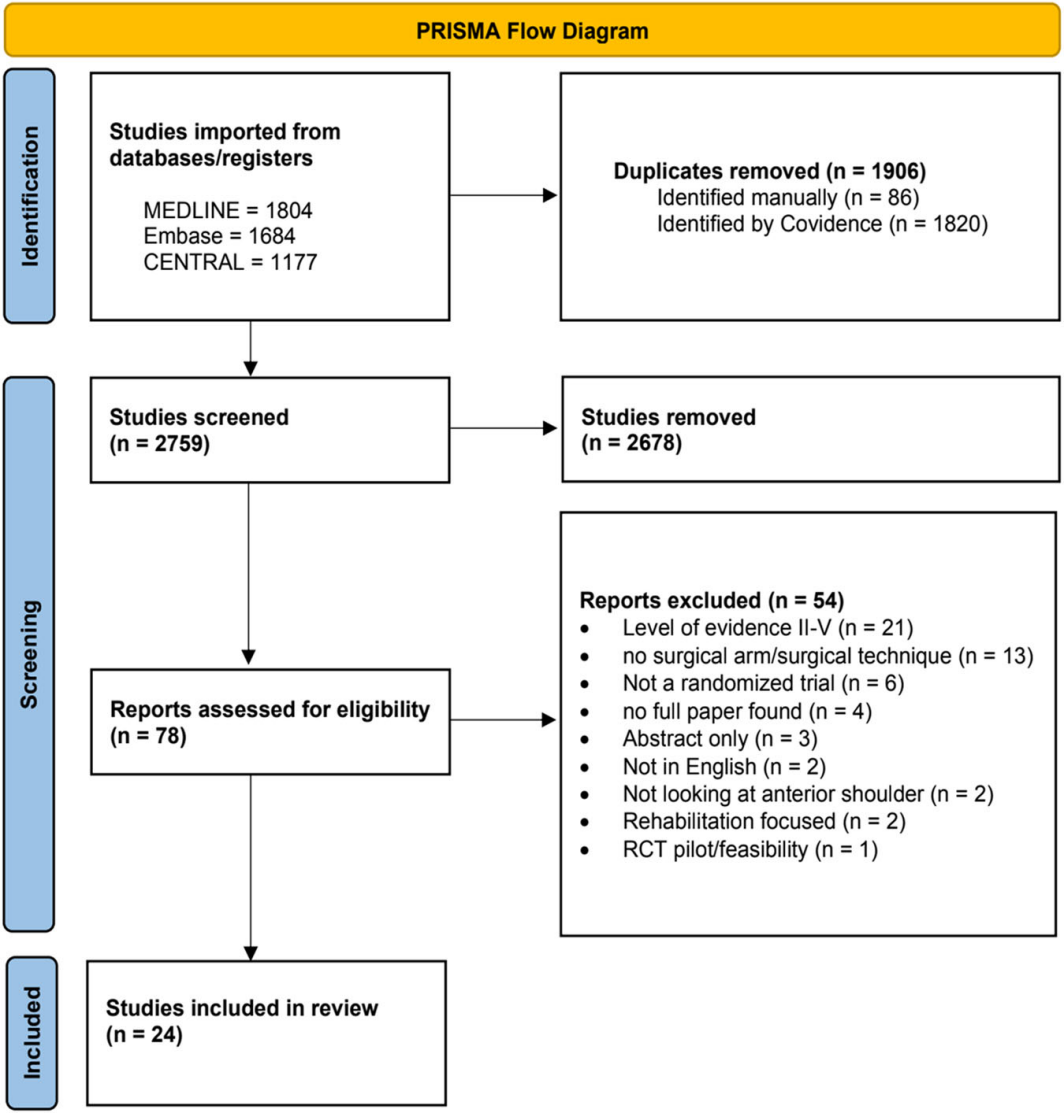
PRISMA flow diagram.

**Figure 2:**
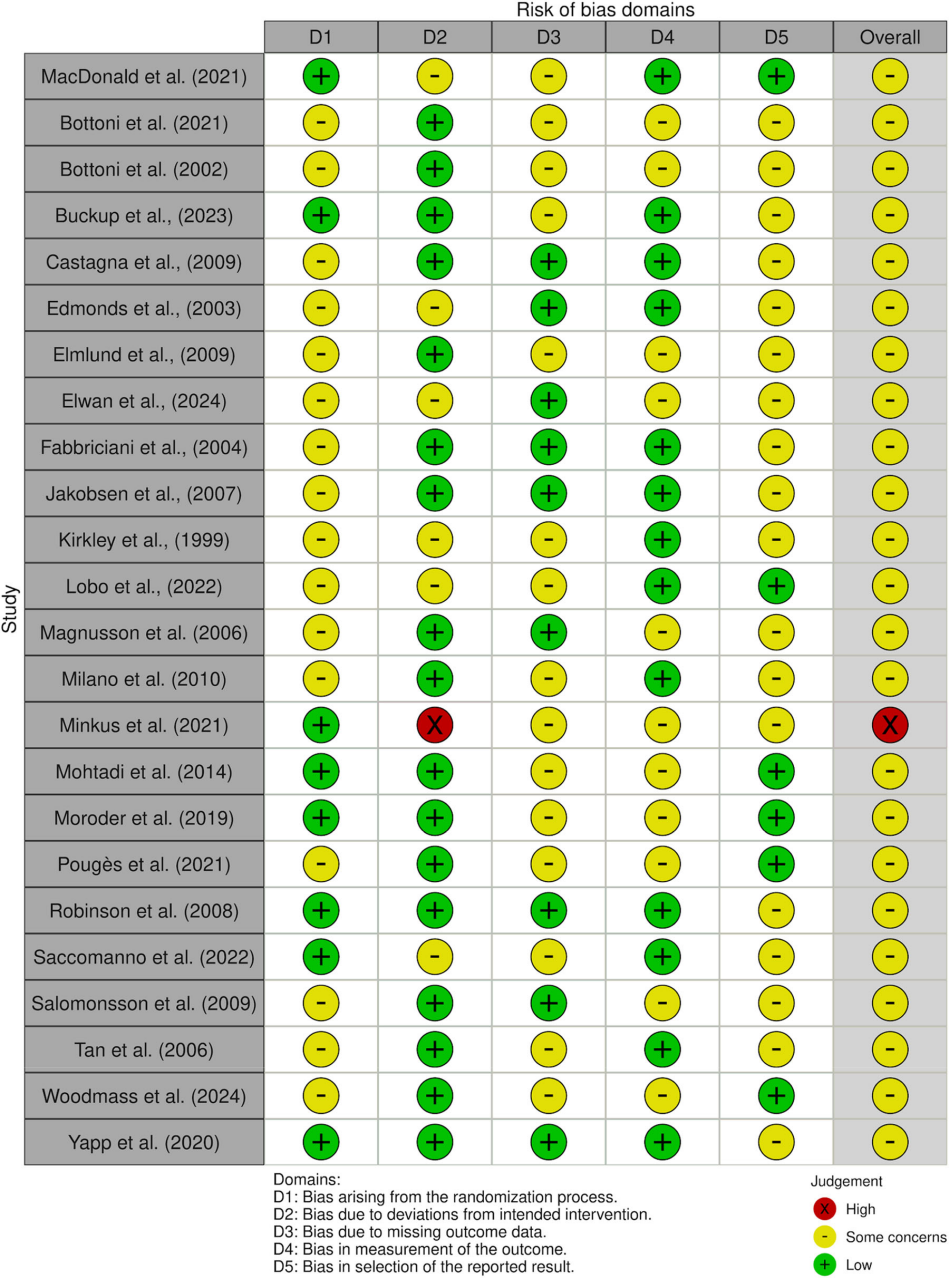
RoB assessment for included studies. RoB, risk of bias.

The search identified 4665 studies, with 2759 studies remaining after all duplicates were removed. A total of 24 studies were included in the final analysis (Figure 1) [8, 9, 10, 13, 19, 20, 21, 23, 32, 34, 39, 40, 41, 46, 47, 48, 49, 54, 56, 57, 58, 61, 65, 66]. Inter‐rater reliability assessments showed moderate agreement at the title/abstract screening stage (*κ* = 0.52) and almost perfect agreement at the full‐text screening stage (*κ* = 0.94). RoB assessments showed overall concern in 23 studies, with only one study having high concern (Figure 2). The average RoB score was 2.25 ± 0.8.

From the included studies, the total number of participants was 1559, with a mean sample size of 65 patients (range 24–162) (Table 2). The mean age across the 24 studies was 26.6 years (range 21.4–40), and the % female on average was 15.1% (range 0%–30%). A total of eleven countries were represented. Canada contributed the largest number of studies (*n* = 5) [19, 34, 40, 48, 65], followed by Italy (*n* = 4) [13, 23, 46, 57] and Sweden [20, 41, 58], the United Kingdom [56, 61, 66], and Germany [10, 47, 49] (*n* = 3 each). The United States contributed two studies [8, 9], while Egypt and Saudi Arabia (combined study) [21], Denmark [32], Brazil [39] and France [54] were represented by one study each. The average number of sites was 1.9 (range 1–13), and the average number of PROMs was 2.1 (range 0–4). The studies were published between 1999 and 2024, with most published in the 2020s (*n* = 10) [8, 10, 21, 39, 40, 47, 54, 57, 65, 66], followed by the 2000s (*n* = 10) [9, 13, 19, 20, 23, 32, 41, 56, 58, 61], the 2010s (*n* = 3) [46, 48, 49] and 1990s (*n* = 1) [34]. The year with the most studies published was 2021 (*n* = 4). In terms of categorization, seven studies assessed outcomes following minor changes to the procedure [10, 20, 39, 41, 46, 57, 61], four assessed outcomes following major changes to the procedure [13, 40, 56, 65], six compared a surgical versus non‐surgical approach [9, 19, 32, 34, 47, 54] and seven compared two procedures against each other [8, 21, 23, 48, 49, 58, 66].

**Table 2:** Included studies' characteristics, trial characteristics and demographics.

Number	Author (year)	Journal (impact factor)	Country	Recruitment period	Number of sites	Age mean (SD) [range]	Sample size	Follow‐up, months [range]	Treatment (intervention/comparator)
1	MacDonald et al. (2021) [40]	*Shoulder and Elbow Surgery Journal* (2.9)	Canada	June 2011 to May 2017, 71 months	2	REMP = 27.3 (8.8) [14.4–53.6]No REMP = 27.8 (8.8) [15.4–55.2]	108 total, 54 per group were randomized REMP = 46 analyzed at 24 months No REMP = 41 anaylzed at 24 months	25.4	Bankart repair with infraspinatus remplissage (*n* = 46, Mean age = 27.3 ± 8.8 [14.4–53.6])
									Bankart repair only w/No remplissage (*n* = 41, Mean age = 27.8 ± 8.8 [15.4–55.2])
2	Bottoni et al. (2021) [8]	*American Journal of Sports Medicine* (4.5)	United States	April 2001 to September 2002, 17 months	1	40 [34–57]	64 total, 32 per group randomized, Arthroscopic group = 28 analyzed at 15 years. Open stabalization = 32 analyzed at 15 years	180 [180–204]	Arthroscopic stabalization (*n* = 28)
									Open stabalization using a deltopectoral approach (*n* = 32) Mean age for both groups = 40 [34–57]
3	Bottoni et al. (2002) [9]	*American Journal of Sports Medicine* (4.5)	United States	November 1994 to April 1998, 41 months	1	22.4Op = 21.6 [19–26]Non‐op = 23 [19–26]	24 total, 3 lost to follow‐up12 for non‐operative treatment9 for operative treatment	36	Arthroscopic stabalization (*n* = 9, Mean age = 21.6 [19–26])
									Stabalization using shoulder immobalization (*n* = 12, Mean age = 23 [19–26])
4	Buckup et al. (2023) [10]	*International Orthopedics* (2.6)	Germany	2014–2019, 72 months	1	Group 1 = 24.2 (6.2)Group 2 = 24.3 (7.2)	53 total, 27 in Group I (2 × 3.5 mm anchors) and 26 in Group II (3 × 2.9 mm anchors)Follow‐up I,Group I = 25, Group II = 24Follow‐up II,Group I = 23, Group II = 22	28.9	2 × 3.5 mm, knotless Bio‐Pushlock Anchor (*n* = 25, Mean age = 24.2 ± 6.2)
									3 × 2.9 mm knotless PEEK‐Pushlock anchor (*n* = 23, Mean age = 24.3 ± 7.2)
5	Castagna et al. (2009) [13]	*Knee Surgery Sports Traumatology and Arthroscopy Journal* (5.0)	Italy	January 2004 to December 2005, 24 months	1	28.2Group A = 29.1Group B = 27.3	40 patients were randomized, 20 per group	24	Group A—Only Anterior capsulorraphy w/suture acnhor technique (*n* = 20, Mean age = 29.1)
									Group B—Two posterior plications before doing anterior capsulorraphy similar to Group A (*n* = 20, Mean age = 27.3)
6	Edmonds et al. (2003) [19]	*Knee Surgery Sports Traumatology and Arthroscopy Journal* (5.0)	Canada	Not reported	1	Surgery group = 20.79 (2.53)[17.05–24.87]Trad group = 21.95 (3.91)[15.47–28.34]	24 total, 11 in surgery group, and 13 in traditional group	19 [3–36]	Arthroscopic Stabalization ‐ Transglenoid suturing tehcnique for treatment of Bankart Lesion followed by immobilization (*n* = 11, Mean age = 20.79 ± 2.53 [17.05–24.87])
									Shoulder Immoblization for 4 weeks (*n* = 13, Mean age = 21.95 ± 3.91 [15.47–28.34])
7	Elmlund et al. (2009) [20]	*American Journal of Sports Medicine* (4.5)	Sweden	Not reported	Not reported	PPLA Group = 26 [16–50]PGA Group = [15–45]	40 were randomized, 20 in each.PPLA, at 7 years 18 went through radiographical assessment and 17 went through clinical assessmentPGA, at 7 years 17 went through radiographical assessment and 16 went through clinical assessment	Median: 80 [64–96]	Bankart reconstruction with Poly‐l‐Lactic acid polymer tacks (*n* = 20, Mean age = 26 [16–50])
									Bankart reconstruction with polygluconate‐B Polymer (PGA) (*n *= 20, Mean age = 30 [15–45])
8	Elwan et al. (2024) [21]	*Journal of Arthroscopy and Joint Surgery* (0.3)	Saudi Arabia and Egypt	December 2020 to May 2023, 29 months	2	Eden Hybinette group = 25.05 (4.57)Latarjet group = 26.5 (5.68)	40 total, 20 per group	At least 12 months	Iliac graft—Eden Hybinette Procedure (*n* = 20, Mean age = 25.05 ± 4.57)
									Laterjet repair (*n* = 20, Mean age = 26.5 ± 5.68)
9	Fabbriciani et al. (2004) [23]	*The Journal of Arthroscopic and Related Surgery* (5.4)	Italy	January 1998 to July 1999	1	Group 1 = 24.5 [19–33]Group 2 = 26.8 [21–30]	60 total, 30 per group	2	Arthroscopic examination followed by SCOI technique (*n* = 30, Mean age = 24.5 [19–33])
									Diagnositc Arthroscopy followed by a deltopectoral approach open repair (*n* = 30, Mean age = 26.8 [21–30])
10	Jakobsen et al. (2007) [32]	*The Journal of Arthroscopic and Related Surgery* (5.4)	Denmark	Not reported	13	Repair group = 23 [15–39]Conservative group = 20 [15–31]	76 total,Repair group = 37Conservative group = 39	120	Open Bankart repair (*n* = 37, Mean age = 23 [15–39])
									Arthroscopy followed by immobilization for 2 days (*n* = 39, Mean age = 20 [15–31])
11	Kirkley et al. (1999) [34]	*The Journal of Arthroscopic and Related Surgery* (5.4)	Canada	Not reported	2	Traditional = 22.75Surgery = 22.1	40 total,Traditional = 21 (only 19 were followed up with)Surgery = 19	33.9	Arthroscopic Stabalization followed by a rehab protocol (*n* = 19, Mean age = 22.7)
									Shoulder immobalization for 3 weeks followed by rehab (*n* = 21, Mean age = 22.1)
12	Lobo et al. (2022) [39]	*The Journal of Arthroscopic and Related Surgery* (5.4)	Brazil	February 2017 to April 2018, 15 months	1	Knotted = 31.2 (10.1)Knottless = 32.6 (11.1)	Total of 64, 32 in each group was randomized.At 6 months,51 total,24 in knotted group27 in knottless group	24	Diagnositic Arthroscopy followed by arthroscopic repair using SutureTak 3.0 mm single loaded anchors (*n* = 24, Mean age = 31.2 ± 10.1)
									Diagnostic Arthroscopy followed by arthroscopic repair using PushLock 2.9 mm knotless anchors (*n* = 27, Mean age = 32.6 ± 11.1)
13	Magnusson et al. (2006)[41]	*The Journal of Arthroscopic and Related Surgery* (5.4)	Sweden	Not reported	1	Polylactic acid polymer (PLLA) = 26 [16–50]PGACP = 30 [15–45]	40 overallPLLA = 20PGACP = 20	25.5	PLLA implants in arthroscopic Bankart reconstruction (*n* =20, Mean age = 26 [16–50])
									Polygluconate co‐polymer implants in arthroscopic Bankart reconstruction (*n* =20, Mean age = 30 [15–45])
14	Milano et al. (2010) [46]	*Knee Surgery Sports Traumatology and Arthroscopy Journal* (5)	Italy	Not reported	1	Metal anchors = 28 [16–43]Biodegradable anchors = 28 [16–52]	70 patients in final evaluationMetal anchors = 36Biodegradable anchors = 34	Median: 24.5 [22–29]	Metal suture anchors in arthroscopic stabilization (*n* =36, Mean age = 28 [16–43])
									Biodegradable suture anchors in arthroscopic stabilization (*n* =34, Mean age = 28 [16–52])
15	Minkus et al. (2021) [47]	*American Journal of Sports Medicine* (4.5)	Germany	October 2011 to October 2017	7	Immobilization = 26.7 (5.8)Surgical = 25.7 (6.2)	112 total patientsImmobilization = 60 (47 F/U)Surgical = 52 (44 F/U)	24	Immobilization in 60° of external rotation and 30° of abduction
									Surgical treatment with arthroscopic shoulder stabilization
16	Mohtadi et al. (2014) [48]	*The Journal of Bone & Joint Surgery* (4.3)	Canada	2001–2008	1	Open repair = 27.8 (7.9) [16–53.7]Arthroscopic repair = 27.2 (9.0) [16.5–59]	226 randomized, 113 per group before exclusionsOpen repair = 97 (79 F/U)Arthroscopic repair = 98 (83 F/U)	24	Open surgical repair
									Arthroscopic stabilization
17	Moroder et al. (2019) [49]	*Journal of Shoulder and Elbow Surgery* (2.9)	Germany	2012–2015	2	Iliac‐crest bone graft transfer (ICBGT) = 29 (9) [18–57]Latarjet = 31 (8) [18–47]	60 randomizedICBGT = 30Latarjet = 30	24	ICBGT
									Latarjet procedure
18	Pougès et al. (2021) [54]	*American Journal of Sports Medicine* (4.5)	France	March 2014 to November 2016	1	Surgical = 21.5 [20.5–22.5]Non‐operative = 21.3 [20–22.5]	40 totalSurgical = 20Non‐operative = 20	24	ABR
									3 weeks of internal rotation immobilization
19	Robinson et al. (2008) [56]	*The Journal of Bone & Joint Surgery* (4.3)	United Kingdom	September 2001 to January 2005	1	24.3 (4.6) ABR25.3 (4.8) arthroscopic lavage only (ALO)Ranges not reported, but eligibility criteria is ages 15–39	88 total, 43 undergoing ABR, 45 undergoing ALO42 patients from each group received final follow‐up	24	ABR
									ALO
20	Saccomanno et al. (2022) [57]	*Knee Surgery, Sports Traumatology, Arthroscopy* (5.0)	Italy	Not reported		[18–47]Knotless = Median: 24.5, IQR: 16.3Knotted = Median: 27, IQR: 10.5	78 patients randomized (7 lost to F/U)Knotless = 39 (34 follow‐up)Knotted = 39 (37 follow‐up)	Median: 44 [36–60]	PEEK knotless suture anchors in arthroscopic capsulolabral repair
									Biodegradable knotted suture anchors in arthroscopic capsulolabral repair
21	Salomonsson et al. (2009) [58]	*Acta Orthopaedica* (2.5)	Sweden	November 1991 to April 1995	1	28 [16–63]Bankart repair = [16–63]Putti–Platt = [17–52]	66, with 62 replying to the 10‐year follow‐upBankart repair = 33Putti–Platt = 33	24	Modified Putti–Platt procedure
									Bankart repair with suture anchors
22	Tan et al. (2006) [61]	*The Journal of Arthroscopic and Related Surgery* (5.4)	United Kingdom	April 2000 to June 2003	1	28 (7) [17–49]Nonabsorbable (G II) = 27 (7) [18–45]Absorbable (Panalok) = 28 (8) [17–49]	130 included, 124 completed study.Nonabsorbable (G II) = 63Absorbable (Panalok) = 61	31.2 [18–60]	ABR with absorbable suture anchors (Panalok)
									ABR with non‐absorbable suture anchors (G II)
23	Woodmass et al. (2024) [64]	*The American Journal of Sports Medicine* (4.5)	Canada	2011–2017	2	REMP = 27.3 (8.8) [14.4–53.6]No REMP = 27.8 (8.8) [15.4–55.2]	108 total, 54 per group were randomizedREMP = 46 analyzed at 24 monthsNo REMP = 41 analyzed at 24 months	49.3 (27.1)	ABR with an arthroscopic infraspinatus remplissage
									ABR alone
24	Yapp et al. (2020) [66]	*The Journal of Bone & Joint Surgery* (4.3)	United Kingdom	September 2001 to January 2005	1	At long‐term follow‐up:ABR = 24.7 [23–26.5]Arthroscopic washout (AWO) = 23.8 [21.8–25.7]	88 total, 43 undergoing ABR, 45 undergoing AWO33 ABR and 32 ALO continued to long‐term follow‐up (65 total)	170.5 [144–192]	ABR
									AWO

Abbreviations: IQR, interquartile range; SD, standard deviation.

### Bibliometric analyses

The studies included were published across eight different journals, with the *Journal of Arthroscopic and Related Surgery* and the *American Journal of Sports Medicine* representing the highest number of included studies (*n* = 6 each). The average impact factor for the included studies was 4.3 (range 0.30–5.40). The median citation density was 4.90 citations per year (range 1.00–19.30), and the mean scholarly score density was 6.75 (range 0.05–24.00). Citation density was the greatest in studies that assessed surgical versus non‐surgical management for ASI, followed by studies that compared two procedures against each other, then studies that assessed major changes to the surgical procedure. Studies that assessed minor changes to surgical procedures had the smallest citation density, although this was not significant but had a large effect size (7.78 ± 3.81, 7.65 ± 6.43, 6.5 ± 4.90 and 2.60 ± 0.96, respectively, *p* = 0.149, *η* ^2^ = 0.23). This trend was similarly observed in the scholarly score density. The average *H*‐index score of the first author was 19.0 (range 1.0–61.0), and the mean *H*‐index of the last author was 23.4 (range 1.0–61.0). The ANOVA showed a significant difference with regard to the first author's *H*‐index between the different study categories. Studies that assessed major changes to surgical procedure had the highest first author *H*‐index, followed by studies that compared two procedures against each other, then studies that assessed surgical versus non‐surgical approach. Studies that assessed minor changes to the surgical procedure had the lowest first‐author *H*‐index (39.5 ± 17.37, 20.14 ± 13.01, 14.33 ± 10.91 and 10.14 ± 9.44, respectively, *p* = 0.008). Detailed results of the ANOVA can be found in Table 3.

**Table 3:** ANOVA results for the different study categories.

Variable	Category 1—Minor changes to surgical procedure (*n* = 7)	Category 2—Major changes to surgical procedure (*n* = 4)	Category 3—Surgical vs. non‐surgical management (*n* = 6)	Category 4—Surgical procedure/technique comparison (*n* = 7)	*p*	*η* ^2^
Citations density	2.60 ± 0.96	6.5 ± 4.90	7.78 ± 3.81	7.86 ± 6.45	0.149	0.237
Scholarly composite score density	2.98 ± 1.87	8.95 ± 8.33	9.28 ± 4.29	9.15 ± 7.92	0.188	0.213
Sqrt(IF)	2.17 ± 0.26	2.03 ± 0.23	2.21 ± 0.10	1.77 ± 0.60	0.241	0.22
Number of PROM	2.57 ± 1.40	1.50 ± 1.29	2.00 ± 1.55	2.00 ± 1.29	0.662	0.075
Total number of outcomes	6.43 ± 4.12	2.50 ± 1.91	3.17 ± 1.94	5.71 ± 3.95	0.175	0.215
Number of trial sites	1.00 ± 0.00	1.50 ± 0.58	4.17 ± 4.92	1.28 ± 0.49	0.126	0.244
Follow‐up means	36.87 ± 20.23	30.43 ± 12.59	42.82 ± 38.36	62.36 ± 77.60	0.690	0.069
Total sample size	62.00 ± 30.89	79.75 ± 28.57	48.83 ± 28.09	73.29 ± 40.03	0.452	0.121
Risk of bias score	2.00 ± 0.57	3.00 ± 0.82	1.83 ± 0.75	2.43 ± 0.79	0.089	0.273
First author *H*‐Index	10.14 ± 9.44	39.5 ± 17.37	14.33 ± 10.91	20.14 ± 13.01	0.008*	0.437
Last author *H*‐index	22.29 ± 4.72	19.75 ± 9.54	32.83 ± 13.70	18.43 ± 22.01	0.333	0.153

Abbreviations: ANOVA, analysis of variance; PROM, patient‐reported outcome measure.

* *p* value < 0.05.

Correlation analysis revealed significant correlation for the first author *H*‐index, which demonstrated significant fair correlation of *r* = 0.45 when correlated with citations per year, and 0.42 when correlated with scholarly composite score per year (*p* = 0.026, and *p* = 0.041, respectively). In addition, RoB scores were fairly correlated with citation density (*r* = 0.43, *p* = 0.036). The rest of the variables demonstrated fair or poor correlation when correlated with citation per year or composite score per year, although this was not significant (Tables 4 and 5).

**Table 4:** Correlation analysis between citation density and mentioned variables.

Variable	*R*	*p*
Sqrt(IF)	0.08	0.710
Year of publication	0.02	0.919
Study design	0.49	0.136
Number of PROM	0.19	0.375
Total number of outcomes	0.16	0.467
Number of trial sites	0.25	0.244
Follow‐up means	0.08	0.725
Total sample size	0.24	0.251
First author *H*‐index	0.44	0.030*
Last author *H*‐index	0.29	0.171
ROB score	0.43	0.036*

Abbreviations: PROM, patient‐reported outcome measure; RoB, risk of bias.

* *p* value < 0.05.

**Table 5:** Correlation analysis between scholarly composite score density and mentioned variables.

Variable	*R*	*p*
Sqrt(IF)	0.02	0.926
Year of publication	0.15	0.480
Study design	0.46	0.179
Number of PROM	0.24	0.261
Total number of outcomes	0.24	0.255
Number of trial sites	0.23	0.291
Follow‐up means	0.06	0.795
Total sample size	0.26	0.216
First author *H*‐index	0.41	0.047*
Last author *H*‐index	0.27	0.207
ROB score	0.38	0.066

Abbreviations: PROM, patient‐reported outcome measure; RoB, risk of bias.

* *p* value < 0.05.

### Altmetrics analyses

Thirteen studies were included for altmetrics analysis [8, 9, 23, 32, 39, 40, 47, 48, 49, 54, 56, 65, 66]. The average Altmetrics attention score was 10.85 (range 1.00–25.00). On average, the included studies had 5.17 online mentions (range 0.00–28.00), with the highest number of mentions on average coming from posts on X (9.38 posts), followed by Facebook posts, Blog/News mentions and Policy/Wikipedia mentions (0.31 posts each). Through those X posts, a total of 353,690 users were reached for an average of 27,206.90 users reached per paper (range 0.00–71,599.00). Finally, across all studies, the median scholarly composite score density was 6.75 mentions per year (range 0.05–24.00).

## DISCUSSION

### Summary of key findings

Analysis showed no significant difference in citation density or scholarly composite score density across the different RCTs categories and other predictors such as journal impact factor, time since publication, study category, number of PROMS, total number of outcome measures, number of sites, follow‐up means, total sample size and last author *H*‐index score. It did, however, show that there was a fair positive correlation between the first author's *H*‐index and citation/scholarly composite density. This significant difference was also noted in the ANOVA, showing that RCTs that had major changes to their surgical procedure or compared two surgical procedures had a higher first author H‐index. In addition, a fair, positive correlation between the study's RoB score and citation density was noted, hinting at a potential relationship between the rigorous design of RCTs and the potential reach of a study.

### Relation to previous literature

An author's *H*‐index summarizes an author's scholarly influence by capturing their productivity (i.e., number of published articles) as well as the citation impact of those articles. As such, an author has an index *h* when *h* of their publications have been cited at least *h* times. In other words, the *H*‐index reflects the volume and impact of an author's published work [30, 31]. Correlation analysis showcased that it is fairly associated with citation and scholarly score density, suggesting that author‐level visibility may alter the dissemination of the management of ASI trial results beyond the content of the trial itself. This pattern is consistent with the Matthew Effect, a concept introduced by Robert Merton in 1968, which describes how prominent authors receive disproportionate visibility for their work due to their established reputation [37, 45]. While theoretical, it can provide a mechanism that explains the fair correlation between increased *H*‐author density and their citation density or even their scholarly composite score. Importantly, however, the *H*‐index does not directly measure methodological quality, and is influenced by career length and other factors such as institutional prestige or multicenter collaborations (despite the non‐significant correlations observed with the latter two variables).

From the 24 studies analyzed, only thirteen (54%) were found to have altmetric data. The sources of bibliometric data were consistent with Evanview et al. and Collins et al. [15, 22], showing that the online activity in orthopaedic research predominantly comes from X and Facebook mentions. Despite this, the systematic review by Evaniew et al. [22] showcased that *Shoulder and Elbow* online mentions had a median of 2 with an interquartile range of 0–8, while this bibliometric analysis's results had an average of 5.6 online mentions. The increase in online mentions likely reflects the increasing integration between traditional journal dissemination (e.g., editorials and conferences) and digital social media platforms (e.g., X and Facebook), although the exact relationship remains unknown

Research published is often assigned a Level of Evidence (LOE), which is a method of classification developed to help appraise scientific research and inform the user of the effectiveness of a particular intervention. For therapeutic research, which investigates the result of a treatment, studies can be assigned a Level of Evidence score ranging from Level I to Level V. Level I studies are often RCTs with narrow confidence intervals and systematic reviews of Level I RCTs, while prospective comparative studies are Level II, Case–control and Retrospective comparative studies are Level III [12]. Prior bibliometric/altmetric analysis on ASI methods of management often pooled study designs (e.g., cohort, cross‐sectional, case‐series and RCTs) together without discriminating against the different levels of evidence [2, 4, 27, 50, 62, 67]. While this has its own advantages, the creation of models that help develop clinical guidelines is increasingly relying on the results stemming from RCTs, which are often regarded as having a higher quality output than other types of studies due to their rigorous criteria and methods of bias control [27, 43]. As such, it is important to clearly identify and isolate the factors influencing the dissemination of results that help shape such guidelines, rather than averaging such findings across designs seen in previous studies. This study reinforces this concept as it was demonstrated that increasing control over the RoB was correlated with a higher citation density and scholarly score density.

Despite this, even when study designs are pooled in previous bibliometric/altmetric research, there does not seem to be a consensus on a study's level of evidence and its higher bibliometric/altmetric impact on the treatment of ASI. For instance, Allahabadi et al. showcased that the top‐cited articles had lower levels of evidence and poorer methodological quality, while O'Dwyer et al. claimed that higher altmetric scores were associated with studies with higher levels of evidence [4, 50]. Isolating the study design in this paper helped better inform the true factors influencing a study's reach, showing that increasing RoB control and author's popularity play a bigger role in the dissemination of study results.

### Strengths and limitations

This study's biggest strength is the inclusion of both bibliometric and altmetric data when trying to quantify the impact of study as it provides a broader and more contemporary perspective on scholarly influence beyond traditional citation counts, citation count reflected how much other researches in the academic world build‐upon this researcher's work, while altmetrics showcased the effect this paper had on a more broader and more public level.

Several limitations should also be acknowledged. First, restricting inclusion to English‐language publications may have excluded relevant RCTs published in other languages. Second, citation counts and altmetric measures, while widely used, are imperfect proxies for research quality or clinical impact, as they can be influenced by journal visibility, geographic trends, or self‐citation. In addition, only thirteen studies were included in Altmetric analyses, which potentially reduces the overall power of the reported altmetric results and correlations. Third, the relatively small number of eligible RCTs in ASI limits the generalizability of observed trends. Finally, the study reflects citation activity only up to the date of our search, and subsequent publications or evolving citation patterns are not captured.

## CONCLUSIONS

In this citation analysis of RCTs studying the management of ASI, first author *H*‐index was the only factor fairly associated with greater citation and scholarly score density. The RoB score was also fairly associated with citation density. Altmetrics analysis did not reveal any significant patterns, suggesting a limited reach beyond academic audiences. As such, it is important to recognize the influence of author‐level visibility and robustness of an RCTs' design to control for bias can have an impact on the dissemination of academic information.

## AUTHOR CONTRIBUTIONS

All authors were involved in conceptualizing and designing the study. Amr AlMasri and Rohaan Syan were involved in the data collection, analysis and drafting of the manuscript. All authors were involved in the review and editing process. The final manuscript was approved for publication by all authors.

## CONFLICT OF INTEREST STATEMENT

The authors declare no conflicts of interest.

## ETHICS STATEMENT

The authors have nothing to report.

## Data Availability

The data that support the findings of this study are available on request from the corresponding author.
